# Expanding the Spectrum of Diabetic Striatopathy: Insights from a Case of Hyperglycemia-Induced Propriospinal Myoclonus

**DOI:** 10.5334/tohm.850

**Published:** 2023-12-29

**Authors:** Debaleena Mukherjee, Subhankar Chatterjee, Peyalee Sarkar, Ritwik Ghosh, Shambaditya Das, Biman Kanti Ray, Alak Pandit, Julián Benito-León, Souvik Dubey

**Affiliations:** 1 Department of Neuromedicine, Bangur Institute of Neurosciences, IPGMER & SSKM Hospital, Kolkata, India; 2 Department of Endocrinology & Metabolism, Medical College & Hospital, Kolkata, India; 3 Department of General Medicine, Burdwan Medical College & Hospital, Burdwan, India; 4 Department of Neurology, University Hospital “12 de Octubre”, Madrid, Spain; 5 Research Institute (i+12), University Hospital “12 de Octubre”, Madrid, Spain; 6 Centro de Investigación Biomédica en Red Sobre Enfermedades Neurodegenerativas (CIBERNED), Madrid, Spain; 7 Department of Medicine, Complutense University, Madrid, Spain

**Keywords:** Diabetes mellitus, diabetic striatopathy, movement disorders, Propriospinal Myoclonus

## Abstract

This video abstract delves into the expanded definition of diabetic striatopathy, linked initially to hyperglycemia-induced choreoballism and striatal hyperintensity on magnetic resonance imaging, but now recognized to encompass a broader range of acute onset, non-choreoballistic movement disorders in diabetes mellitus, including tremors, hemifacial spasm, parkinsonism, different types of myoclonus, dystonia, restless leg syndrome, ataxia, and dyskinesias. We report the case of a 45-year-old female patient with type-2 diabetes mellitus who developed propriospinal myoclonus, characterized by painless, involuntary jerky movements of the bilateral lower limbs in a supine position after admission for suspected rhino-orbital mucormycosis. The abnormal movements resolved entirely following the control of her blood glucose levels, suggesting a direct correlation between hyperglycemia and the clinical picture. This case highlights the importance of considering a wide range of differential diagnoses for abnormal lower limb movements in diabetic patients, emphasizing the need for accurate identification of movement semiology, routine bedside capillary blood glucose checks, and prompt hyperglycemia management to resolve such movement disorders effectively.

## Background

Diabetic striatopathy (DS) is classically defined as hyperglycemia associated with either acute onset choreoballism and/or striatal hyperintensity on T1-weighted magnetic resonance imaging (MRI) (or hyperdensity on non-contrast computed tomography) [1,2]. However, this definition of DS seems to be narrowed as our knowledge about the spectrum of acute onset movement disorders in diabetes mellitus is increasingly expanding. In this sense, the most extensive clinical series depicting the clinical-radiological spectrum of acute onset movement disorders in diabetes mellitus (N = 59 patients) showed that the majority (55.9%) had no MRI changes [3]. Due to this fact, our group has recently proposed a classification of DS that includes symptomatic DS (striatal neuroimaging lesions in association with a clinically evident movement disorder and hyperglycemia), clinically isolated DS (clinically evident movement disorders without striatal changes in neuroimaging), and radiologically isolated DS [4].

Authors herein report a novel case of propriospinal myoclonus (PSM) as a complication of hyperglycemia. This case delves into the expanded definition of DS, now recognized to encompass a broader range of acute onset, non-choreoballistic movement disorders in diabetes mellitus, including tremors, hemifacial spasm, parkinsonism, different types of myoclonus, dystonia, restless leg syndrome, ataxia, and dyskinesias [1,2,3].

## Phenomenology Shown

A 45-year-old female patient was admitted with suspected rhino-orbital mucormycosis. She had a history of SARS-CoV-2 infection four months earlier when she was also diagnosed with type-2 diabetes mellitus. Since the first night of admission, she developed abnormal involuntary jerky movement involving bilateral lower limbs, particularly during the supine position, suggesting PSM (video 1). Capillary blood glucose (CBG) was 540 mg/dl then. Other metabolic parameters (including electrolytes, blood gas analysis, blood ketone, renal, and liver panels) were normal. Glycated hemoglobin was 14%. Brain MRI and magnetic resonance angiography (MRA) were normal. Following blood glucose control with a basal-bolus insulin regimen, abnormal movements completely disappeared in the next two days.

**Video 1:** Shows a 45-year-old female patient with painless involuntary jerky movements involving bilateral lower limbs in the supine position.

## Educational Value

PSM, a relatively rare hyperkinetic movement disorder, is characterized by painless, flexor arrhythmic axial jerks involving the lower half of the body, aggravated by sensory cues and appearing after assuming a supine position [5].

In the context of DS, acute hyperglycemia can shift central nervous system metabolism towards anaerobic pathways, resulting in a depletion of γ-aminobutyric acid (GABA), an inhibitory neurotransmitter [1,2,3,4]. This reduction in GABA levels could diminish the inhibitory control over neuronal firing [1,2,3,4]. With reduced GABAergic inhibition, there would be an increased excitability in the spinal cord neurons, particularly in the propriospinal tracts. This increased excitability could lead to spontaneous and exaggerated reflex responses that propagate along the propriospinal pathways, which connect different spinal cord levels and result in the characteristic jerky movements of PSM [5].

Apart from PSM, other conditions may present with lower limb abnormal movements:

- Paraballism: This condition is, however, of higher frequency and with a greater amplitude.
- Restless leg syndrome: It is usually associated with pain or uncomfortable sensation with a solid temporal relationship with sleep.
- Paroxysmal kinesigenic dyskinesia: Here, the abnormal movements occur during walking.
- Paroxysmal non-kinesigenic dyskinesia: In this case, the frequency of the attack rate is much lesser than observed in our case.
- Flexor spasm: This condition is generally painful with other signs of upper motor neuron lesions. Besides, neuroimaging was not suggestive of stroke due to angioinvasion by mucor. Notably, it is worth noting that the patient was admitted to a “mucor ward” and, therefore, a spinal MRI could not be performed. Brain MRI brain and MRA could be performed as part of the management protocol in a “mucor ward” since our primary concerns were cranial angioinvasion by mucormycosis. Moreover, overnight recovery of the abnormal lower limb movements by correcting hyperglycemia established the solid temporal association of PSM with hyperglycemia, making spinal MRI unnecessary in that clinical context.
- Bilateral limb shaking transient ischemic attack: This condition occurs while standing, and, in this case, MRA was also normal.
- Psychogenic or functional movement disorders: This condition was ruled out as the abnormal movements persisted even during deviation of attention or absence of observers. Abnormal movements in our case were without any clinical symptomatic variability over the disease course and ceased only with the correction of the hyperglycemic state. However, electrophysiological studies would best differentiate psychogenic axial jerks from true PSM [6]. Unfortunately, this could not be arranged in a mucormycosis ward due to logistic restrictions.
- COVID-19-induced movement disorders- This was not considered possible due to a lack of temporality in events (between the history of SARS-CoV-2 infection and the onset of movement disorder), normal neuroimaging, and the presence of another plausible alternative explanation (i.e., hyperglycemia) [7].

This case highlights the importance of considering a wide range of differential diagnoses for abnormal lower limb movements in diabetic patients, emphasizing the need for accurate identification of movement semiology, routine bedside CBG checks, and prompt hyperglycemia management to resolve such movement disorders effectively.
